# Supplementary material for: Effect of sensor number and location on accelerometry-based vertical ground reaction force estimation during walking
Source: PLOS Digit Health. 2024 May 14;3(5):e0000343. doi: 10.1371/journal.pdig.0000343 (PMC11093287; doi:10.1371/journal.pdig.0000343)
Supplement: Attachment — Submitted filename: Response to Reviewers_v2.docx [file pdig.0000343.s001.docx]

# Response to Reviewers

Additional Editor Comments (if provided):

**I find the study to be relevant and well-organized. However, I concur with our reviewers that there are areas requiring clarification, particularly in the methods section. I would like to request a major revision, as suggested by our reviewers. Their comments to the authors contain specific questions that need to be addressed in the revision.**

We thank the editor and the invited reviewers for providing additional comments to improve the quality of our manuscript. We outline specific revisions in our responses that follow.

**In my role as a Guest Editor for the journal, I'd like to highlight the importance of diversity in digital health research. If the study primarily involved young and healthy volunteers, it's crucial to discuss how the results might translate to patients with Osteoarthritis (OA), who typically differ in age, body weight, and health status. While you've mentioned this in the limitations, elaborating on the specific characteristics of patients with OA and how they may impact the model's performance is essential.**

We agree entirely with the editor. We have now elaborated on how different characteristics of people with OA may influence model accuracy on lines 293-300:

“One limitation is that this study only includes young, healthy participants. Thus, our models may not extend to individuals with pathologic gait or who may have or be at risk for developing OA. We specifically included an underloading (-5%) condition in our protocol that emulates the less dynamic vGRF profiles (lower values in early and late stance, higher values during midstance, Fig. 5A,E,I) commonly exhibited by individuals with knee OA.(2,29,30) This additional underloading condition serves as an early step towards generalizability in our models by including inputs similar to those from the target population. Nevertheless, future studies will be needed to include individuals with knee OA who typically differ from our control group by age, body mass, health status, and other demographics.”

**In addition to the changes recommended by our reviewers, please consider the following minor revisions:

Line 200: Referring to Fig. 5G, the hashtag (#) should be placed in the bar representing estimated values to demonstrate the comparison with measured values.**

We have updated the figure and caption (lines 427-431) to account for this edit.

**“Figure 5:** We further explore the three most accurate sensor configurations, analyzing the Waist (A-D), Foot-Waist (E-H), and Shank-Waist (I-L) models across each of the loading conditions (underloading=blue, control=black, overloading=red). Asterisks indicate a significant difference from the control condition for that measurement type, whereas hashes (#) represent a significant difference between estimated (open boxes) and measured (filled boxes) vGRF loading peak.“ **Line 206: Referring to Fig. 5K, the same applies here. The # should be positioned over the bar for measured values to signify significant differences with the measured values.**

Changed as recommended. See similar comment and response directly above. **Line 264: You mentioned using the "acceleration vector magnitude" and combining the 3-dimensional components into one composite acceleration measure. Please ensure that this is clearly explained in the methodology.**
We have clarified our explanation of this calculation in the revised methods on lines 156-159:

”To remove any bias of sensor orientation and sidedness, we calculated the acceleration vector magnitude (or Euclidian norm) as: $Acceleration Vector Magnitude= \sqrt[2]{x^{2}+ y+ z^{2}}$, where x, y, and z, are the 3D orthogonal components of the acceleration measurement.”

**Line 275: You discussed that the “interpretability plot reveals some of the underlying features that our model may use to estimate the vGRF.” Please provide a clearer explanation of Fig. 6, both in the results section and the captions. Pay particular attention to the side graphs of mean-normalized summed weights.**

We thank the reviewer for pointing out an area requiring further clarity. In the revised results we have added a few sentences prior to our descriptive narrative of Figure 6 (lines 224-234):

“Figure 6 explores characteristics of the multilayer perceptron model for the Waist-only sensor configuration. We show a heatmap of the model coefficient weights for the input (W_Acc_, vertical axis) and output (vGRF, horizontal axis). In the sub-plots, we also show the mean-normalized average weights across each instance of the gait cycle (black bars) and the average input W Acc and output vGRF signals (gray line).

The estimated vGRF (horizontal axis and top sub-axis) demonstrated strong positive weights during early (5-15% GC) and late (50-60% GC) stance phase, as well as strong negative weights during leg swing (60-100% GC). Although the vGRF weights during midstance (15-50% GC) included both positive and negative values, those weights were relatively smaller. Model weights for the input Waist acceleration vectors (vertical axis and right sub-axis) were more complicated. However, we tended to see more positive weights during the stance phase (0-60% GC) and more negative weights during the swing phase (60-100% GC). “

We also updated our Figure caption to now read (lines 433-445):

**“Figure 6:** To address interpretability of the Waist model, we show the resultant model coefficient weights for the input (W Acc, vertical axis) and output (vGRF, horizontal axis) layers of our multilayer perceptron neural network model. Model weights are shown via heatmap as 1% instances of the gait cycle (%GC), along each axis from top-left to bottom-right, with higher positive weights in red, lower negative weights in blue, and zero weights in black. Along each axis, we show the mean-normalized summed weights for each column/row as a bar graph, with higher relative weights shown above or to the right of the axes. Because the W_Acc_ is plotted along the vertical axis, the W_Acc_ weights are shown to the right, aligned with the vertical axis. Similarly, the estimated vGRF is plotted along the horizontal axis of the heatmap, thus the vGRF weights are shown on top, in line with the horizontal axis. The black bars represent the mean-normalized neural net weight for that row (W_Acc_, vertical axis) or column (Avg vGRF, horizontal axis) at that instance of the gait cycle. For additional context, we also overlay the average Waist acceleration vector magnitude (Avg W_Acc_) along the vertical axis and the average estimated vGRF (Avg vGRF) along the horizontal sub-axis, both shown in gray.”

**I believe that addressing these points will significantly enhance the quality and clarity of your paper.**

We thank the editor for their attention to detail and thoughtful comments.

Reviewers' comments:

**Reviewer #1:**

**The work is interesting and give some important information about the vGRF. However I have some questions regarding the methods and the way the authors take the data, because is not clear in the paper. What is the biofeedback used, how is measured (I guess using the treadmill but is not clear)?
Is the information in the screen part of the bio feedback?**

We appreciate the reviewer’s thoughtful comments. We have now elaborated on our biofeedback protocol in the revised methods on lines 130-137:

“Specifically, participants performed two additional five-minute walking trials while responding to real-time biofeedback driven by instantaneous force measurements from the instrumented treadmill. Visual biofeedback was designed to prescribe, in randomized order, 5% higher and 5% lower loading peak vGRF (overloading and underloading, respectively) during the first 50% of the stance phase (Figure 1A). Participants viewed a screen showing two bar plots, representing their left and right vGRF loading peak, updated in real time as the average from the two previous steps (Figure 1A). As displayed in Figure 1A, we instructed participants to match their peak vGRF (blue bars updated on every step) with the target force (horizontal red line) to the best of their ability.”

**The EMG sensors (Delsys Trigno) are used to collect information, but later is not clear how this information is used to compare with accelerometers. Because the model is not mentioned, is not clear if the sensors include EMG or just are IMU (Not only accelerometers).**

We apologize for any confusion with the data collection protocol. We have updated this section to now read (lines 121-124):

“We used Delsys Trigno (Natick, MA, USA) sensors not to collect electromyographic recordings, but to collect bilateral three-dimensional acceleration from the dorsum of each foot, the anterior/medial bony surface of the distal shank, and posteriorly on the waist (Figure 1A). The version of our Trigno sensors did not include gyroscope or magnetometer measurements.

**It seems that acelerometers are only compared with the treadmill information. Basically, the methods section need to be improved, in order to better understand the work and its results.**

The reviewer is correct in their interpretation here. We only used the acceleration signals from the Delsys Trigno sensors, and our analyses compared ground-truth vGRF measurements from the instrumented treadmill to vGRF estimates from models trained on acceleration signals. We refer the reviewer to the preceding comments and our revision of the text described above where we have updated our methods section to better clarify our methods. We appreciated the opportunity to make this clearer.

**The training of the neural networks used needs to be explained, because is not clear the relation between the trained model using the treadmill and the accelerometer. Are the same or only this training was used with the acelerometers?**
We thank the reviewer for this opportunity to clarify our neural network training scheme. We have added the following in the revised methods on lines 165-169:

“We trained the MLP neural networks to estimate the ground truth vGRF signal recorded via instrumented treadmill as a 100-point vector using a Nx100-point accelerometer input corresponding to the sensor(s) used (1x100 for a single sensor, 2x100 for two sensors, etc.). These 100-point signals represent the vGRF and accelerometer recordings interpolated to percentage of the gait cycle (%GC), measured between consecutive heel strikes during walking.”

**Reviewer #2:**

**This is a well conducted study that extended previous work on using wearable sensors to estimate ground reaction force. The novelty of this study lies in the systematic investigation of various sensor configurations on the estimation of ground reaction force during walking. The manuscript was well written. The methods are appropriate overall, but I have one comment on the methods:**

**1.      I understand that both the left and right GRFs and accelerations (50 strides from each side) were combined together for training and testing of machine learning model. Have the authors looked at the analysis using only one side of data to build machine learning model for each side separately? Would it be possible that this way of analysis will show that the other configuration of sensors might actually perform better than waist only model? This is because that there might be asymmetry in steps of the healthy participants, which makes the waist model being advantageous than other configurations overall. However, this advantage might change if data from one side was used separately, especially in participants with larger asymmetry. It might also be worth checking using statistics whether asymmetry had any effect on the error of the models. This may be relevant to the clinical application of the model as asymmetry is often observed on patients with OA.**

We thank the reviewer for this comment. In line with this interpretation, our idea was to train the model using unilateral data (from both left and right sides) specifically because of the potential for asymmetries in movement patterns. By using the acceleration vector magnitude, we created resultant acceleration profiles for all 3 segments measured (foot, shank, & waist). These acceleration profiles were included as separate steps within our training/testing dataset, so although both left and right sides are included, they are recorded as distinct steps (no laterality).

Although additional study would be warranted, we tend not to believe that separate left and right models would improve the accuracy of the estimation models. We do agree that it would be beneficial to quantify the asymmetry level of the tested sample, and thus we have done so in the plot below. We depict the loading peak for all 6000 steps in our sample (3000 each side) and show no evidence for asymmetry at least in terms of vGRF loading peak. In future iterations of this work, the presence of osteoarthritis may motivate us to classify symptomatic versus asymptomatic limbs during the training and testing process.
