# Supplementary material for: Effect of sensor number and location on accelerometry-based vertical ground reaction force estimation during walking
Source: PLOS Digit Health. 2024 May 14;3(5):e0000343. doi: 10.1371/journal.pdig.0000343 (PMC11093287; doi:10.1371/journal.pdig.0000343)
Supplement: Attachment — Submitted filename: Response to Reviewers 2.docx [file pdig.0000343.s002.docx]

# Response to Reviewers Round 2

Journal Requirements:
Please review your reference list to ensure that it is complete and correct. If you have cited papers that have been retracted, please include the rationale for doing so in the manuscript text, or remove these references and replace them with relevant current references. Any changes to the reference list should be mentioned in the rebuttal letter that accompanies your revised manuscript. If you need to cite a retracted article, indicate the article's retracted status in the References list and also include a citation and full reference for the retraction notice.

**We have refreshed our citations and have found no retractions in our references via the Zotero plug-in tool.**

Additional Editor Comments (if provided):

The paper has improved substantially. Please address the minor comments provided by one of the reviewers. Thank you.
**We thank the editorial team in helping to ensure a quality manuscript for publication.**

Review Comments to the Author

Please use the space provided to explain your answers to the questions above. You may also include additional comments for the author, including concerns about dual publication, research ethics, or publication ethics. (Please upload your review as an attachment if it exceeds 20,000 characters)

Reviewer #1: Authors have addressed all the comments and corrected the issues. The paper is highly improved and very clear now. The contribution is important.

**We thank the reviewer for their thoughtful contributions to improve this paper.**

Reviewer #2: Thanks for authors’ response. It is clear from the provided data that there was no asymmetry in the study sample. Therefore, I would agree with the authors that separating left and right might not improve the accuracy of the model. However, I would suggest make this clear in the conclusion that this model is only for health participants with little or no asymmetry. Please see specific suggestions below:

**We thank the reviewer for their thoughtful contributions to improve this paper.**

Page 2, line 39. Abstract:  the last sentence may be written as “Our results suggest that a system designed to monitor changes in walking vGRF or to deploy targeted biofeedback may only need a single accelerometer located at the waist for healthy participants.”

**We have updated this sentence as suggested.**

Page 3 line 55: Author summary: the last sentence may be written as “Our results suggest that a system designed to monitor changes in walking vGRF or to deploy targeted biofeedback may only need a single accelerometer located at the waist for healthy participants.”

**We have updated this sentence as suggested.**

Page 11 line 246: Discussion: the last sentence may be written as “Our results suggest that a system designed to monitor changes in walking vGRF or to deploy targeted biofeedback may only need a single accelerometer located at the waist for healthy participants.”

**We have updated this sentence as suggested.**

Page 14 line 309-315: the conclusion may be written as “We can accurately estimate the vGRF loading peak within 5% of body weight and distinguish between biofeedback-induced loading conditions in healthy participants using only a waist accelerometer by implementing a multilayer perceptron neural network model. ….. We suggest that a wearable sensor system comprised of a single waist sensor is not only sufficient, but optimal for implementing a biofeedback-based therapy system to prescribe limb loading during gait retraining in healthy participants.”

**We have updated this sentence as suggested.**
